# Photoplethysmogram beat detection using Symmetric Projection Attractor Reconstruction

**DOI:** 10.3389/fphys.2024.1228439

**Published:** 2024-02-26

**Authors:** Callum Pettit, Peter H. Charlton, Philip J. Aston

**Affiliations:** ^1^ Department of Mathematics, University of Surrey, Guildford, United Kingdom; ^2^ Department of Public Health and Primary Care, University of Cambridge, Cambridge, United Kingdom

**Keywords:** photoplethysmography, beat detection, symmetric projection attractor reconstruction, PPG signals, attractor, poincaré section, inter-beat intervals

## Abstract

Many methods have been proposed to detect beats in photoplethysmogram (PPG) signals. We present a novel method which uses the Symmetric Projection Attractor Reconstruction (SPAR) method to generate an attractor in a two dimensional phase space from the PPG signal. We can then define a line through the origin of this phase space to be a Poincaré section, as is commonly used in dynamical systems. Beats are detected when the attractor trajectory crosses the Poincaré section. By considering baseline drift, we define an optimal Poincaré section to use. The performance of this method was assessed using the WESAD dataset, achieving median *F*_1_ scores of 74.3% in the Baseline phase, 63.0% during Stress, 93.6% during Amusement, and 97.7% during Meditation. Performance was better than an earlier version of the method, and comparable to one of the best algorithms identified in a recent benchmarking study of 15 beat detection algorithms. In addition, our method performed better than two others in the accuracy of the inter-beat intervals for two resting subjects.

## 1 Introduction

Photoplethysmogram (PPG) signals are now widely measured both by consumer smartwatches and by clinical pulse oximeters for unobtrusive physiological monitoring. A key step in many PPG signal processing tasks is detecting heartbeats in the PPG signal: this can form the basis for heart rate monitoring; it is fundamental to extracting inter-beat intervals (IBIs) from which to assess autonomic function through pulse rate variability analysis (Mejía-Mejía et al., 2020); and is often a precursor to estimating blood pressure from PPG pulse wave morphology (Mejía-Mejía et al., 2022). Therefore, accurate PPG beat detection algorithms are of the utmost importance in the field.

**FIGURE 1:**
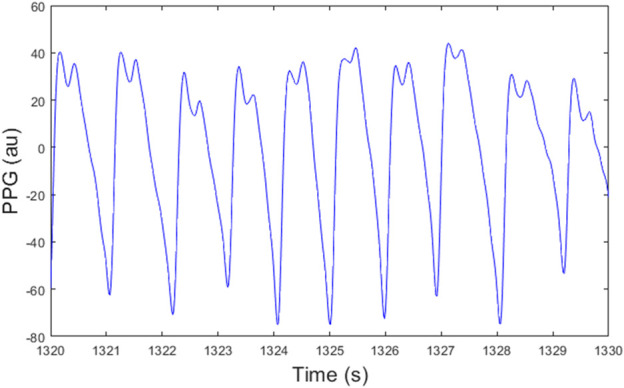
A PPG signal for subject S4 with double peaks.

Beat detection in photoplethysmogram (PPG) signals is not a straightforward task. First, PPG signals are highly susceptible to noise and in particular motion artifact. Second, PPG signals do not exhibit a prominent feature indicating a heartbeat, in contrast to electrocardiogram (ECG) signals which contain a high-frequency R-wave each heartbeat. Third, the morphology of PPG signals can vary from one beat to the next. PPG beats are commonly detected using some form of either peak or trough detection (Charlton et al., 2022). However, peaks or troughs in the PPG are not always clearly defined, particularly in the presence of noise. Furthermore, PPG signals sometimes exhibit two peaks per heartbeat, with the first being the highest for some beats and the second being the highest for other beats (see Figure 1), which can introduce timing errors into beat detection.

In this study we propose a novel beat detection algorithm for PPG signals based on the generation of Symmetric Projection Attractor Reconstruction (SPAR) attractors (Aston et al., 2018; Lyle and Aston, 2021). The SPAR method derives attractors from signals using a number of equally spaced points running through the signal to generate a bounded attractor in a two-dimensional phase space. In this work, we use a SPAR attractor as previously defined and combine it with the concept of a Poincaré section, a line which cuts through the attractor that is a concept borrowed from dynamical systems theory (Wiggins, 2003), to detect beats from SPAR attractors. The beat detection algorithm presented in this paper has been refined since an early version of the method was presented in Charlton et al. (2022). Thus, we present new results on the performance of the SPAR beat detector and compare its performance with the best methods in Charlton et al. (2022).

Our novel beat detection algorithm may be applied not only in heart rate monitoring, but also for applications requiring accurate inter-beat interval estimation such as heart rhythm assessment, heart rate variability analysis, and stress monitoring. The algorithm may be particularly useful for wearable devices such as smartwatches, smart rings, and earbuds, due to its low computational complexity and its ability to handle baseline wander.

Heart Rate Variability (HRV) analysis (Acharya et al., 2006) consists of deriving a range of useful measures from the IBIs, which are obtained from the distances between successive R peaks of an ECG signal. Similarly, Pulse Rate Variability (PRV) (Mejía-Mejía et al., 2020) performs a similar analysis using the IBIs derived from a PPG signal. It has been concluded that PRV is a valid surrogate for HRV for healthy, resting subjects (Mejía-Mejía et al., 2020). Thus, we also compare the accuracy of the SPAR beat detector IBIs and two other PPG beat detection algorithm IBIs against the ECG RR intervals for two resting subjects which have no artefacts in their PPG signals.

## 2 Methods

### 2.1 Dataset

The dataset used in this study was the Wearable Stress and Affect Detection (WESAD) dataset from the UC Irvine Machine Learning Repository (Schmidt et al., 2018). This dataset includes: i) PPG signals recorded from an Empatica E4 wrist-worn device at a sampling frequency of 64Hz; and ii) ECG signals recorded simultaneously from a RespiBAN chest-worn device at 700Hz. For each of the 15 subjects there is around 100 min of data. Each subject followed a protocol including five phases: Baseline, Amusement, Stress, Meditation 1, and Meditation 2. We considered all phases in our analysis.

### 2.2 Symmetric Projection Attractor Reconstruction

**FIGURE 2:**
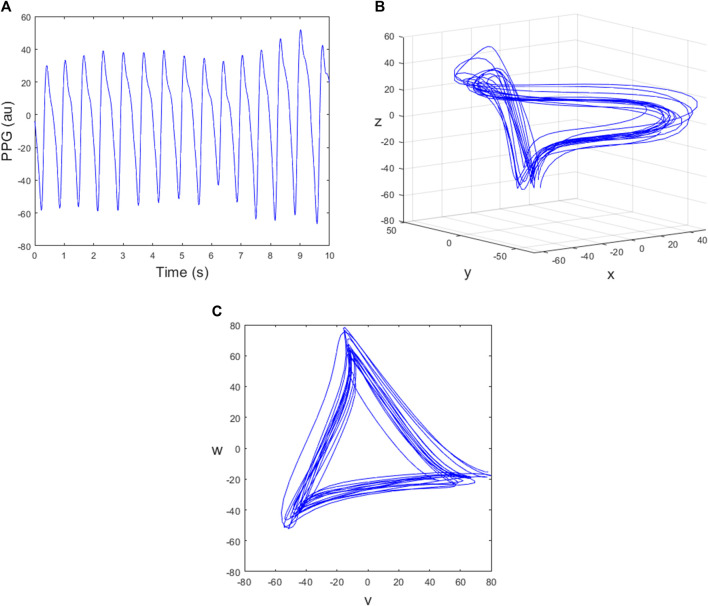
The steps used to generate a two-dimensional attractor using the SPAR method. The data is 420–430 s of subject 13. **(A)** A sample of PPG signal; **(B)** The trajectory plotted in three dimensional phase space; **(C)** The corresponding attractor in the (v,w) plane.

The Symmetric Projection Attractor Reconstruction (SPAR) method converts the PPG signal, which is measured over time, into an attractor in a bounded domain, by plotting its trajectory in three-dimensional phase space and then projecting it onto a particular plane, as described in Aston et al. (2018). The process of obtaining an attractor from a PPG signal is summarised in Figure 2, and the remainder of this section provides further mathematical details.

Using Takens’ method for reconstructing attractors using time delay coordinates (Takens, 1981), an attractor can be reconstructed in an *N*-dimensional phase space from a single signal *x*(*t*) by using a vector of delay coordinates given by

$$\left[x\left(t\right),x\left(t-\tau \right),x\left(t-2\tau \right),\ldots ,x\left(t-\left(N-1\right)\tau \right)\right]$$

where *τ* > 0 is a fixed delay and *N* ≥ 2 is the embedding dimension. The embedding dimension was initially chosen as *N* = 3 for ease of visualisation in Aston et al. (2018) although this was later generalised to any embedding dimension *N* ≥ 3 in Lyle and Aston (2021).

For the case *N* = 3, we define the new variables

$$y\left(t\right)=x\left(t-\tau \right),\;\;\;z\left(t\right)=x\left(t-2\tau \right)$$

where the time delay *τ* is chosen to be one-third of the average cycle length (Aston et al., 2018). The trajectory can then be plotted in a three-dimensional phase space as shown in Figure 2B. Projecting the three-dimensional attractor onto a plane perpendicular to the vector (1,1,1) reduces the effect of baseline wander and gives a two-dimensional attractor with approximate three-fold rotational symmetry (Aston et al., 2018). For this, we define the new variables

$$\begin{array}{lll} u & = & \frac{1}{3}\left(x+y+z\right)\\ v & = & \frac{1}{\sqrt{6}}\left(x+y-2z\right)\\ w & = & \frac{1}{\sqrt{2}}\left(x-y\right) \end{array}$$

and then the (*v*, *w*) plane is perpendicular to the vector (1,1,1), (Aston et al., 2018). The resulting attractor in the (*v*, *w*) plane is shown in Figure 2C where the trajectory runs in a clockwise direction and each loop of the attractor corresponds to one cycle of the PPG data.

### 2.3 Using a Poincaré section for beat detection

**FIGURE 3:**
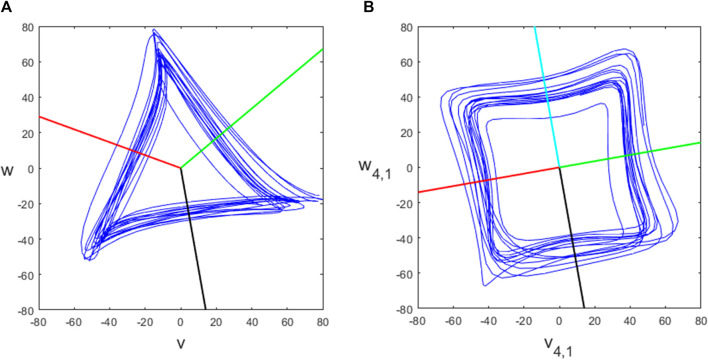
The **(A)** *N* = 3 and **(B)** *N* = 4 attractors and chosen Poincaré sections.

In dynamical systems, described by a system of ordinary differential equations, a Poincaré section that intersects the flow in phase space transversely can be used to convert a continuous dynamical system into a discrete dynamical system (Wiggins, 2003). Here we are working with data, not a dynamical system, but we similarly define a Poincaré section that intersects our attractor as a means of detecting individual beats. The section should be transversal to the flow ideally, which of course can never be guaranteed for data-defined trajectories. Due to the approximate three-fold symmetry of the attractor, there are three natural choices of the Poincaré section, as shown by the black, red and green lines in Figure 3A.

In practice, we define a fixed Poincaré section by the horizontal line *w* = 0, *v* > 0 and rotate the attractor appropriately. The timestamps at which the section is crossed are found by checking the conditions *w*_*i*_ > 0 and *w*_*i*+1_ < 0. Note that these conditions also specify the direction of crossing, namely, from above to below the line. When these conditions are satisfied, linear interpolation is used to find the time t* of the crossing more accurately and then the further condition *v* (t*) > 0 is also checked. If required, IBIs can be obtained by finding the difference between consecutive times at which the attractor intersects a particular Poincaré section.

### 2.4 Varying the embedding dimension *N*

**FIGURE 4:**
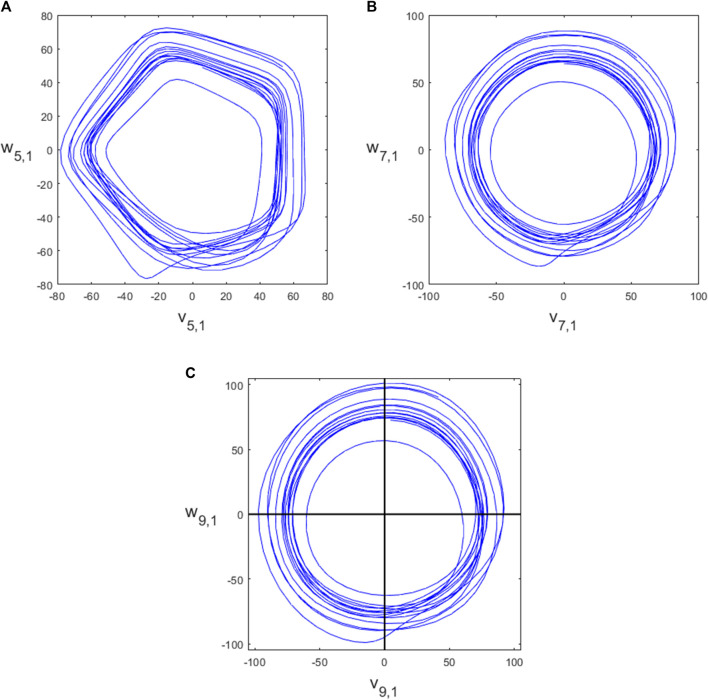
The attractors for **(A)** *N* = 5, **(B)** *N* = 7, **(C)** *N* = 9.

The description of the SPAR method for generating attractors (in Section 2.2) considered an embedding dimension *N* = 3. More generally, the embedding dimension can be set to any integer *N* ≥ 3. Lyle and Aston (2021) showed how to generate similar two-dimensional attractors (*v*_*N*,*k*_, *w*_*N*,*k*_), but with multiple attractors for each dimension *N* > 4 which we call projections, indexed by *k* = 1, … , ⌊(*N* − 1)/2⌋. The (*N*, *k*) attractor has an approximate *m*-fold rotational symmetry where *m* = *N*/gcd (*N*, *k*). The attractor for the PPG signal shown in Figure 2A for *N* = 4 is shown in Figure 3B and for *N* = 5, 7, 9 is shown in Figure 4, all with projection *k* = 1. We investigated using higher projections *k* > 1 but found that they were not suitable as the attractors were more variable in these cases. Therefore all of our examples use projection *k* = 1. Similarly to the *N* = 3 case, we used 4 equally spaced Poincaré sections in approximately the middle of each side of the *N* = 4 attractor, as shown in Figure 3B, to give multiple estimates for individual beats and IBIs.

For higher dimensions, the attractors become more circular in nature (Lyle and Aston, 2021) which is an advantage. However, if *N* is too large, there may be very few data points between each of the delay coordinates if the sampling frequency of the data is relatively low and so there may be a mismatch between the expected positions of the delay coordinates and data points available.

### 2.5 Finding the optimal Poincaré section

**FIGURE 5:**
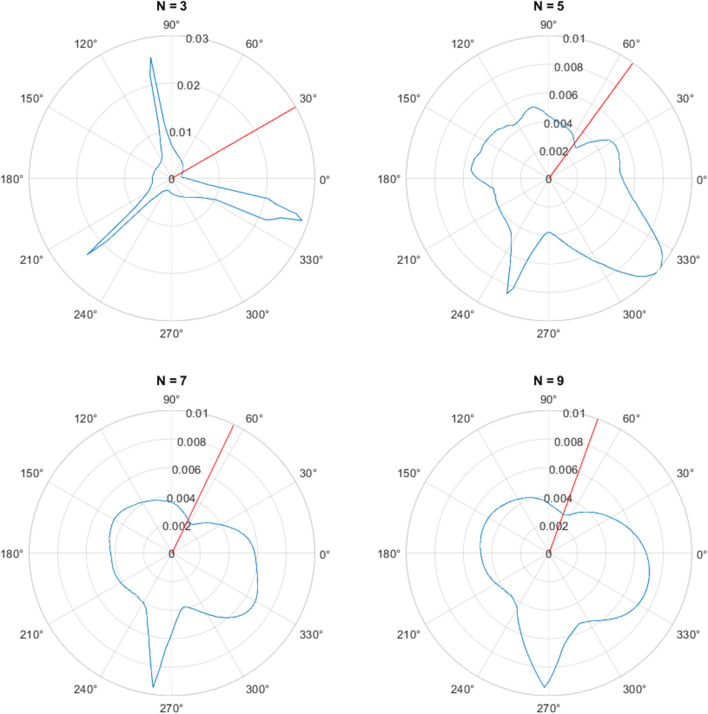
The mean absolute error of the inter-beat intervals for varying angle of the Poincaré section (in degrees) applied to the PPG signal shown in Figure 2A for the attractors with **(A)** *N* = 3, **(B)** *N* = 5, **(C)** *N* = 7, **(D)** *N* = 9. The optimal angle of Supplementary Theorem S1 is depicted by the red lines.

We now consider whether there is an angle for the Poincaré section that consistently gives the best accuracy. An example when finding the errors for the section at all possible angles for the *N* = 3 attractor is shown in Figure 5, where the distance from the centre of the plot indicates the mean absolute error. As might be expected, the errors are high if the section is positioned at the corners of the attractor and is much lower when positioned in the middle of the straight sides of the attractor. However, this pattern is not repeated for higher values of *N*, when the attractor becomes more circular, as is also shown in Figure 5. The optimal angle seems to be always in the first quadrant and has an increasing angle as *N* increases.

We conjecture that the optimal angle of the section is related to baseline wander in the signal and so we consider the effect of linear baseline drift on the attractor by considering the function

$$x\left(t\right)=x_{0}\left(t\right)+ct$$ (1)

where *x*_0_(*t*) is an approximately periodic function which has linear drift added to it, where *c* is a constant.

When *N* = 3, the *v* attractor coordinate is given by

$$\begin{array}{lll} v\left(t\right) & = & \frac{1}{\sqrt{6}}\left(x\left(t\right)+x\left(t-\tau \right)-2x\left(t-2\tau \right)\right)\\ & = & \frac{1}{\sqrt{6}}\left(x_{0}\left(t\right)+x_{0}\left(t-\tau \right)-2x_{0}\left(t-2\tau \right)+ct\right.\\ & & \left.+\;c\left(t-\tau \right)-2c\left(t-2\tau \right)\right)\\ & = & v_{0}\left(t\right)+\frac{3}{\sqrt{6}}c\tau \end{array}$$

where *v*_0_(*t*) is the *v* coordinate generated from the signal *x*_0_(*t*). Similarly, the *w* attractor coordinate in this case is given by

$$\begin{array}{lll} w\left(t\right) & = & \frac{1}{\sqrt{2}}\left(x\left(t\right)-x\left(t-\tau \right)\right)\\ & = & \frac{1}{\sqrt{2}}\left(x_{0}\left(t\right)-x_{0}\left(t-\tau \right)+ct-c\left(t-\tau \right)\right)\\ & = & w_{0}\left(t\right)+\frac{1}{\sqrt{2}}c\tau \end{array}$$

where *w*_0_(*t*) is the *w* coordinate generated from the signal *x*_0_(*t*).

Thus, the baseline drift added to the signal results in a translation of the (*v*_0_, *w*_0_) attractor which is proportional in magnitude to the slope *c* of the drift. The direction of movement in the (*v*, *w*) plane is in a direction *θ* defined by

$$\tan\theta =\frac{c\tau /\sqrt{2}}{3c\tau /\sqrt{6}}=\frac{1}{\sqrt{3}}$$

which is independent of *c*. In this case, we have *θ* = *π*/6 if *c* is positive or *θ* = −5*π*/6 if *c* is negative. The angle *θ* = *π*/6 is shown as a red line on the *N* = 3 plot in Figure 5 and it can be seen that this is very close to the optimal angle in this case.

We can generalise this result to any value of *N* ≥ 3.

Supplementary Theorem S1.

*The* (*N*, *k*) *attractor for* *k* = 1 … , ⌊(*N* − 1)/2⌋ *generated from an approximately periodic signal* *x*_0_(*t*) *with superimposed linear drift given by* Eq. 1 *is related to the* (*N*, *k*) *attractor generated from the signal* *x*_0_(*t*) *only by a translation in the direction* *θ*_*N*,*k*_ *where*

$$\theta_{N,k}=\frac{\pi }{2}-\frac{\pi k}{N}\;\left(c>0\right),\;-\frac{\pi }{2}-\frac{\pi k}{N}\;\left(c<0\right)$$

*The magnitude of the shift is proportional to the slope* *c* *of the drift but the direction of the shift is independent of* *c*.

The proof of this result is given in the Supplementary Material.

We note that as *N* → *∞* for fixed *k*, *θ*_*N*,*k*_ → ±*π*/2. Also, for *k* = 1 and *N* = 3, 5, 7, 9, we have *θ*_*N*,*k*_ = 30°, 54°, 450/7 = 64.29°, 70° respectively. These angles are shown as red lines on the respective plots in Figure 5 and give good agreement with the minimum error in each case.

We conjecture that the angle given in Supplementary Theorem S1 is optimal because baseline drift on the signal moves the attractor in the direction of the section and so has no effect on the time for each cycle whereas, if the direction of translation is not aligned with the section, the IBIs will be increased or reduced by the translation of the attractor.

### 2.6 Algorithm implementation

The SPAR beat detector was implemented as follows. Signals were segmented into 20 s windows with a 5 s overlap. For each window, the time delay parameter *τ* was found by: i) filtering the signal using a fourth order Chebyshev II filter to remove baseline wander, as recommended in Liang et al. (2018) ii) identifying the average cycle length (*i.e.*, the average IBI); as the cycle length which produced the maximum autocorrelation value for the filtered signal (limited to a search between and 0.4 s and 1.5 s, corresponding to a heart rate range of 40–150 bpm); and iii) calculating *τ* as 1/*N* times this average cycle length. The attractor trajectory was then generated from the raw PPG signal using this value for *τ*. Individual beats were then detected as the times at which the attractor trajectory crossed the optimal Poincaré section as given in Supplementary Theorem S1. Multiple embedding dimensions were investigated.

A further step was included to account for noisy signals. The autocorrelation function provides values between −1 and 1, with 1 indicating perfect correlation. Through a manual inspection, a threshold of the maximum autocorrelation value of 0.4 was identified to distinguish between clean and noisy signals. Windows with values of <0.4 were deemed noisy, and so the window was divided in half and the cycle length corresponding to the highest maximum of the autocorrelation function from the two-halves of the window was used as the average cycle length. This worked well where there was high quality signal at the start or end of the window.

In the periods of overlap between consecutive 20 s windows, beats were duplicated but were not necessarily identified at the exact same times, due to a difference in the time delay *τ* for the two windows. The smallest error between the duplicated beats was found and this beat was the last one used from the earlier window before swapping over to the beats in the new window.

Following beat detection, missed or false beat detections were corrected as follows. Missed beats were detected by checking for IBIs greater than the median IBI plus a tolerance of 0.35IBI. The number of missing beats was determined from the IBI divided by the median IBI and the corresponding number of evenly spaced beats was inserted. Similarly, if two beats were closer than the median IBI minus a tolerance of 0.3IBI then the beat closest to its other neighbour was removed.

### 2.7 Performance assessment

The performance of the SPAR beat detector was assessed in three ways: i) PPG-derived IBIs and reference ECG-derived IBIs were compared for short time intervals for illustrative purposes; ii) the accuracy of beat detection was assessed against reference beats identified in the ECG signal using the *F*_1_ score and iii) PPG IBIs were compared with ECG-derived IBIs for two subjects during the Meditation 1 phase. These three approaches are now described.

First, IBIs obtained from PPG signals using the SPAR beat detector were compared to reference IBIs obtained from ECG signals. To do so, beats were detected in PPG signals using the SPAR beat detector, and then IBIs were calculated as the time differences between consecutive beat detections. R-peaks were detected in the ECG signals, manually checked, and corrected where necessary (for the whole dataset). ECG-derived IBIs were then calculated as the time differences between consecutive R-peaks. Performance was expressed as the mean absolute error between PPG- and ECG-derived IBIs.

Second, the performance of the SPAR beat detector was assessed against reference ECG beats using the *F*_1_ score, which is the harmonic mean of the recall (*i.e.*, sensitivity, the proportion of ECG beats which were correctly detected in the PPG) and the precision (*i.e.*, positive predictive value, the proportion of PPG beat detections which were correct). The *F*_1_ score simplifies in this context to

$$F_{1}\left(\%\right)=\frac{2n_{\text{correct}}}{n_{\text{PPG}}+n_{\text{ECG}}}\times 100$$

where *n*_correct_ is the number of correct beats, *n*_PPG_ is the number of PPG beats and *n*_ECG_ is the number of ECG beats. The number of correct PPG beats was determined as in Charlton et al. (2022) by finding the nearest PPG beat to each ECG R peak and designating it as correct if the absolute time difference between the two was <150 ms. The results were reported as median (lower - upper quartiles) *F*_1_ scores on a per subject basis. The performance of the present SPAR beat detector algorithm was compared with that of an earlier version of the algorithm, which was described in Charlton et al. (2022).

It was important to synchronise the timings of ECG and PPG signals since they were measured using different devices. To do so, PPG beats were aligned with ECG R-peaks using the time delay which resulted in the highest number of correct beats (with candidate time delays of between −10 s and 10 s in steps of 10 ms investigated).

Finally, we compared PPG-derived IBIs found using the SPAR beat detector with the ECG-derived IBIs and did a similar comparison with the qppg and MSPTD beat detection algorithms, which were the top two methods for beat detection in Charlton et al. (2022). The sequences of IBIs derived from the ECG and PPG signals can only be compared if they have detected the same number of beats. Also, if the PPG beat detector missed a beat which has then been estimated, this beat is likely to have a larger error which will dominate any errors from correctly detected beats, thus distorting the results. Thus, we chose subjects from the WESAD data using the Meditation 1 phase for which there were no noisy intervals in the signal so that all the beats are detected by the PPG beat detectors. In particular, we chose subjects S9 and S10. (The exception to this was the qppg beat detector which incorrectly detected a few beats at the start for subject S9 and so for this case, we excluded the first 14 s of data, which should have little influence on the results.) The Meditation 1 phase for both of these subjects was 400 s long and consisted of 475 beats for subject S9 and 501 beats for subject S10. The IBIs were found using the three PPG beat detectors (using *N* = 3, … , 10 for the SPAR beat detector) and from the corresponding ECG signals. Each sequence of PPG-derived IBIs was aligned with the ECG-derived IBIs by finding the minimum of the mean absolute error with a shift of ±5 IBIs. We then report the mean absolute error of these aligned sequences of IBIs.

## 3 Results

### 3.1 Illustrative examples

**FIGURE 6:**
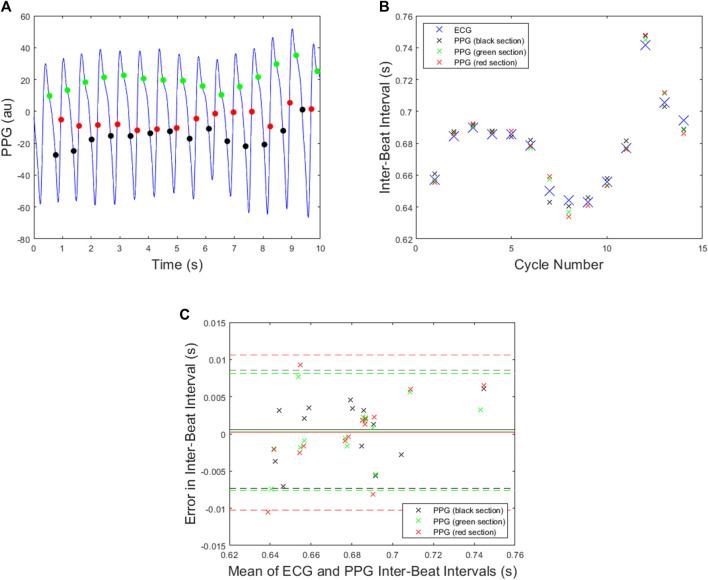
All plots are for the PPG data shown in Figure 2A and the *N* = 3 attractor. **(A)** The beats detected when the delay coordinate x lies on either the green, black or red sections shown in Figure 3A. **(B)** The ECG inter-beat intervals together with the PPG inter-beat intervals for each section. **(C)** A Bland Altman plot for each section with mean indicated by solid lines and mean plus/minus 1.96 x standard deviation indicated by dashed lines.

Illustrative examples of PPG beat detection using the SPAR beat detector are now presented. Figure 6A shows the PPG beats detected using an *N* = 3 attractor for the signal in Figure 2A, where the points shown are the position of the leading delay coordinate, *x*, when the attractor trajectory crosses each of the three sections. The corresponding IBIs obtained are shown in Figure 6B while the Bland Altman plot in Figure 6C shows the errors when compared to the ECG-derived IBIs. This plot shows that the mean of the errors is smallest for the red and green sections, while the standard deviation is the smallest for the black and green sections, thus suggesting that the results obtained from the green section are the best. The mean absolute error for each section is given in Table 1 from which we can see that in this particular case the best results were also obtained for the green section.

**TABLE 1:** The mean absolute error for the inter-beat intervals of the PPG signal shown in Figure 2A.

*N*	Section	Mean absolute error (s)
3	black	0.0036
3	green	0.0031
3	red	0.0039
4	black	0.0045
4	green	0.0031
4	cyan	0.0045
4	red	0.0041

**FIGURE 7:**
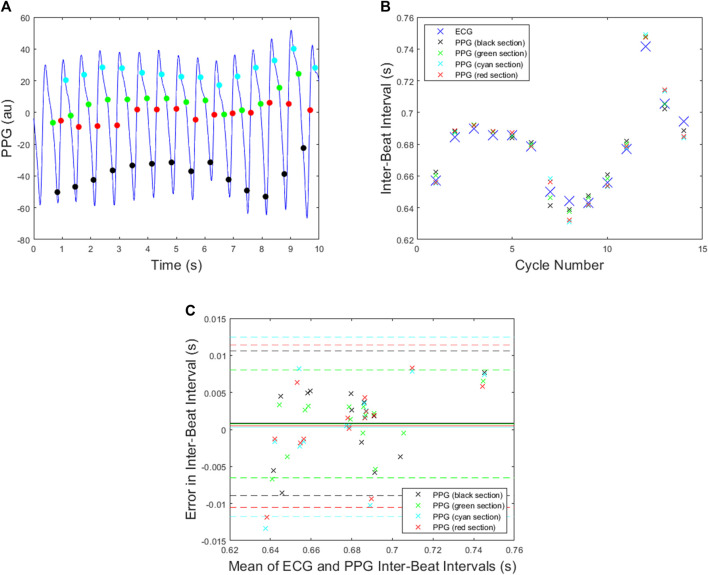
All plots are for the PPG data shown in Figure 2A and the *N* = 4 attractor. **(A)** The beats detected when the delay coordinate x lies on either the green, black, red or cyan sections shown in Figure 3A. **(B)** The ECG inter-beat intervals together with the PPG inter-beat intervals for each section. **(C)** A Bland Altman plot for each section with mean indicated by solid lines and mean plus/minus 1.96 x standard deviation indicated by dashed lines.

In the case of *N* = 4, similar results are shown in Figure 7, and the corresponding mean absolute errors are shown in Table 1. The green section again gave the best results with the smallest standard deviation in the Bland Altman plot and the same error as for the green section with *N* = 3.

### 3.2 The accuracy of the SPAR beat detection

**FIGURE 8:**
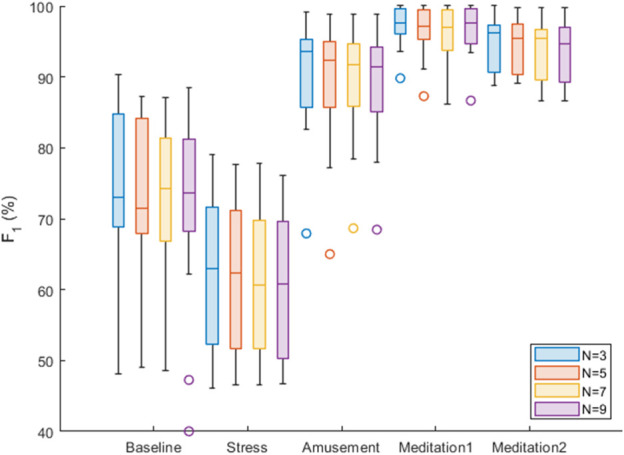
Box plots of the *F*_1_ scores for each of the five conditions and for *N* = 3,5,7,9.

Results relating to the accuracy of the SPAR beat detector are now presented, expressed as the *F*_1_ score. Accuracy was assessed over each of the five protocol phases, and using a range of attractor embedding dimensions: *N* = 3, 5, 7, 9. Box plots were used to summarise these results for all the subjects, as shown in Figure 8. There was only a small difference in the median *F*_1_ scores for the different values of *N*. The best median score was obtained with *N* = 3 for three of the five phases (Stress, Amusement and Meditation 2). *N* = 3 also provided good performance in the other phases, being the second best performer in the ‘Meditation 1’ phase, and the third best performer in the ‘Baseline’ phase (where the median *F*_1_ score with *N* = 3 was 1% lower than that of the best performer, *N* = 7).

**FIGURE 9:**
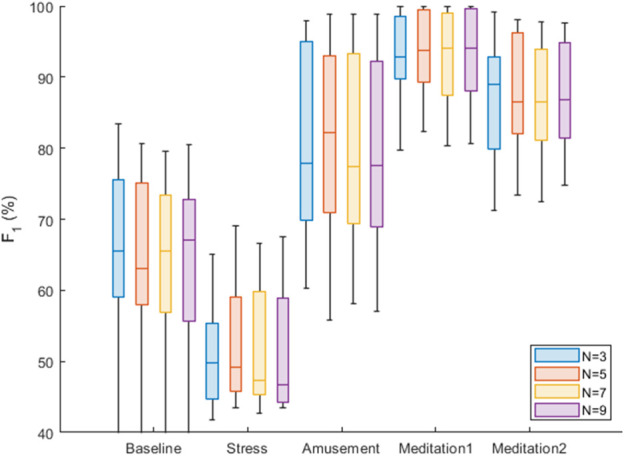
Box plots of the *F*_1_ scores for each of the five conditions and for *N* = 3, 5, 7, 9 using the earlier SPAR method that was used in Charlton et al. (2022).

For comparison, results obtained using the previously reported SPAR method are shown in Figure 9. It can be seen that the new SPAR method gives significantly improved results compared with the previous method.

### 3.3 The accuracy of the SPAR inter-beat intervals

**FIGURE 10:**
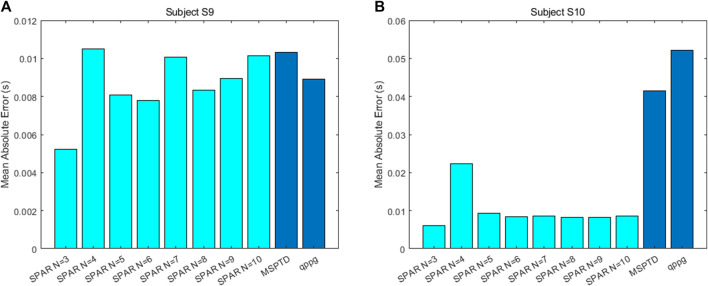
The mean absolute error between the ECG-derived IBIs and the PPG derived IBIs for **(A)** subject S9 and **(B)** subject S10 during the Meditation 1 phase.

The mean absolute error between the ECG-derived IBIs and the PPG-derived IBIs was found for the SPAR, MSPTD and qppg beat detectors for subjects S9 and S10 during the Meditation 1 phase. These errors are shown in Figure 10.

**FIGURE 11:**
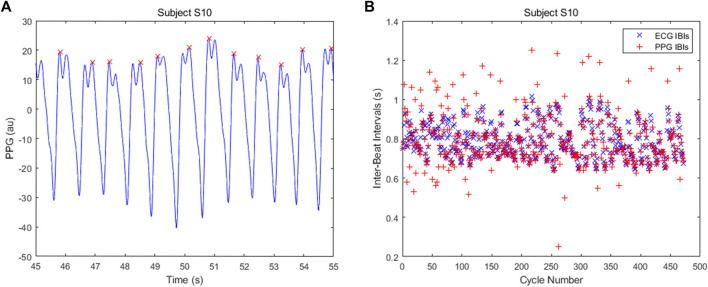
**(A)** Some of the detected beats showing alternation between which of the double peaks is the highest and **(B)** the IBIs calculated by the qppg algorithm for subject S10.

For subject S10, the SPAR errors are much smaller than those for the other two algorithms. The reason for this is that there are quite a few double peaks in this PPG signal and the second peak is sometimes the highest, resulting in inaccurate IBIs. A sample of the signal with the detected peaks and the IBIs calculated using the qppg algorithm are shown in Figure 11. The results for the MSPTD method are similar. We also note that the error for the SPAR method with *N* = 4 is much larger than the other SPAR errors. The reason for this is that this method misses one beat in approximately the middle of the data, and so the second half of the IBIs are out of synch with the ECG IBIs. For the SPAR method, the smallest mean absolute error of 0.0061 was obtained for *N* = 3.

There is no problem with double peaks for subject S9, but here also the SPAR method with *N* = 3 has the lowest mean absolute error of 0.0052 as compared to 0.0103 for MSPTD and 0.0089 for qppg.

The simplest variability metric that is derived from the IBIs is the standard deviation, which is usually referred to as SDNN. The percentage error between the ECG-derived and PPG-derived SDNN is shown in Table 2 for both subjects S9 and S10.

**TABLE 2:** Percentage errors in calculating SDNN compared to the ECG-derived value for subjects S9 and S10.

	S9 (%)	S10 (%)
SPAR (*N* = 3)	2.22	2.16
MSPTD	8.52	42.67
qppg	4.59	45.39

## 4 Discussion

This study has presented a novel algorithm for detecting beats in the PPG signal which is not based on peak detection. The algorithm involves representing the PPG signal as an attractor in phase space using the SPAR method, and identifying beats as the times at which the attractor trajectory intersects the optimal Poincaré section. The algorithm showed good performance on a dataset of PPG signals acquired from a wrist-worn device across different phases of a mental stress protocol.

We now compare our results with those reported in Charlton et al. (2022) for the WESAD dataset and in particular, with the qppg algorithm from the prior study. For the ‘Baseline’ phase, the median *F*_1_ score for qppg was 74.2%, in comparison to the SPAR beat detector’s performance of 74.2% (*N* = 7). For ‘Stress’, the SPAR beat detector achieved 62.6% (*N* = 3), which was a little worse than qppg which achieved 68.7%. For ‘Amusement’, the SPAR beat detector achieved 93.6% (*N* = 3) which was marginally better than the 92.8% achieved by qppg. For ‘Meditation 1’, the SPAR beat detector achieved 97.7% (*N* = 9) which is slightly lower than the 98.3% obtained by qppg. The ‘Meditation 2’ data was not considered in Charlton et al. (2022) so a comparison is not possible in this case. In summary, the new SPAR methodology performed similarly to qppg, which was one of the two best-performing algorithms identified in Charlton et al. (2022). We note that there are some differences in the algorithm assessment methodologies used between these studies, introducing some uncertainty into the above comparison. Nonetheless, in this study the SPAR beat detector demonstrated a clear improvement in performance when compared to an earlier version of the SPAR beat detector.

The calculation of the IBIs for the SPAR method with *N* = 3 outperformed both the MSPTD and qppg algorithms, which were the top two best performing algorithms in Charlton et al. (2022), for two subjects during the Meditation 1 phase. Poor results were obtained for the IBIs for subject S10 due to the regular occurrence of a second peak being the highest, but the SPAR method also performed best for subject S9 where there were no such problems. In the calculation of SDNN, the SPAR method with *N* = 3 had an error of just 2.2% relative to the ECG-derived value for both subjects, whereas the other methods had an error in excess of 40% for subject S10, due to the double peaks, but also had higher errors (8.5% for MSPTD and 4.6% for qppg) for subject S9. Thus, we conclude that the SPAR method is clearly the best performing of the methods considered for calculating the IBIs that are required for HRV/PRV analysis.

In conclusion, the novel SPAR PPG beat detection algorithm presented in this study was found to perform well with wrist PPG signals. The algorithm methodology differs greatly from previously proposed PPG beat detection algorithms as it does not depend on detection of peaks or troughs in the signal but instead calculates times at which the orbit in reconstructed phase space intersects a Poincaré section. These intersections typically occur well away from the peaks and troughs and so are not dependent on the clarity of the peaks and troughs. Future work should investigate how the algorithm performs across different subject groups. Future work may also consider extending the method to detect beats in other cardiovascular signals such as the electrocardiogram, and also to detect events in any approximately periodic data, such as breaths in respiratory signals.

## Data Availability

Publicly available datasets were analyzed in this study. This data can be found here: https://archive.ics.uci.edu/ml/datasets/WESAD+%28Wearable+Stress+and+Affect+Detection%29 WESAD dataset.
