# Supplementary material for: Photoplethysmogram beat detection using Symmetric Projection Attractor Reconstruction
Source: Front Physiol. 2024 Feb 26;15:1228439. doi: 10.3389/fphys.2024.1228439 (PMC10926197; doi:10.3389/fphys.2024.1228439)
Supplement: Supplementary file 1 [file DataSheet1.PDF]

Supplementary Material

1 PROOF OF THEOREM 1

THEOREM 1.
The (N, k) attractor for $k = 1 \ldots, \lfloor (N-1)/2 \rfloor$ generated from an approximately periodic signal $x_0(t)$ with superimposed linear drift given by

$$x(t) = x_0(t) + ct$$

is related to the (N, k) attractor generated from the signal $x_0(t)$ only by a translation in the direction $\theta_{N,k}$ where

$$\theta_{N,k} = \frac{\pi}{2} - \frac{\pi k}{N} \ (c > 0), \ \ -\frac{\pi}{2} - \frac{\pi k}{N} \ (c < 0)$$

The magnitude of the shift is proportional to the slope c of the drift but the direction of the shift is independent of c.

PROOF.
We define the delay coordinates

$$x_{N,j}(t) = x(t - j\tau), \quad j = 0, \ldots, N-1$$

The general v attractor coordinate for any (N, k) pair is given by (Lyle and Aston (2021))

$$\begin{aligned} v_{N,k}(t) &= -\frac{\sqrt{2}}{\sqrt{N}} \sum_{j=0}^{N-1} \cos(2\pi(j+1)k/N) x_{N,j}(t) \\ &= v^0_{N,k}(t) - \frac{\sqrt{2}c}{\sqrt{N}} \sum_{j=0}^{N-1} \cos(2\pi(j+1)k/N)(t - j\tau) \\ &= v^0_{N,k}(t) - \frac{\sqrt{2}ct}{\sqrt{N}} \sum_{j=0}^{N-1} \cos(2\pi(j+1)k/N) + \frac{\sqrt{2}c\tau}{\sqrt{N}} \sum_{j=0}^{N-1} j \cos(2\pi(j+1)k/N) \end{aligned}$$

where $v^0_{N,k}(t)$ is the v coordinate generated from the signal $x_0(t)$. We express the cosine terms in exponential form as

$$\cos(2\pi(j+1)k/N) = \frac{1}{2}\left(e^{2\pi i(j+1)k/N} + e^{-2\pi i(j+1)k/N}\right)$$

Therefore, considering first the sum, we obtain

$$\sum_{j=0}^{N-1} \cos(2\pi(j+1)k/N) = \frac{1}{2} \sum_{j=0}^{N-1} (e^{2\pi i(j+1)k/N} + e^{-2\pi i(j+1)k/N})$$

Both of the exponential terms give rise to a geometric progression which can be summed giving

$$\begin{aligned}\sum_{j=0}^{N-1}(e^{2\pi i(j+1)k/N}+e^{-2\pi i(j+1)k/N}) &= \frac{e^{2\pi ik/N}(1-e^{2\pi ik})}{1-e^{2\pi ik/N}}+\frac{e^{-2\pi ik/N}(1-e^{-2\pi ik})}{1-e^{-2\pi ik/N}} \\ &= 0\end{aligned}$$

since $e^{2\pi ik}=e^{-2\pi ik}=1$ and the numerators are non-zero for the given range of k. For the second sum, we obtain

$$\sum_{j=0}^{N-1} j\cos(2\pi(j+1)k/N)=\frac{1}{2}\sum_{j=0}^{N-1} j(e^{2\pi i(j+1)k/N}+e^{-2\pi i(j+1)k/N})$$

We note that

$$\begin{aligned}\sum_{j=0}^{N-1} jx^j &= x\frac{d}{dx}\sum_{j=0}^{N-1}x^j \\ &= x\frac{d}{dx}\left(\frac{1-x^N}{1-x}\right) \\ &= \frac{x(1+(N-1)x^N-Nx^{N-1})}{(1-x)^2}\end{aligned}$$

Thus

$$\begin{aligned}\sum_{j=0}^{N-1} je^{2\pi i(j+1)k/N} &= e^{2\pi ik/N}\sum_{j=0}^{N-1} j\left(e^{2\pi ik/N}\right)^j \\ &= e^{4\pi ik/N}\left(\frac{1+(N-1)e^{2\pi ik}-Ne^{2\pi ik(N-1)/N}}{(1-e^{2\pi ik/N})^2}\right) \\ &= e^{4\pi ik/N}\left(\frac{1+(N-1)-Ne^{-2\pi ik/N)}}{(1-e^{2\pi ik/N})^2}\right) \\ &= Ne^{4\pi ik/N}\left(\frac{1-e^{-2\pi ik/N}}{(1-e^{2\pi ik/N})^2}\right)\end{aligned}$$

Therefore

$$\begin{aligned}\frac{1}{2}\sum_{j=0}^{N-1} j(e^{2\pi i(j+1)k/N}+e^{-2\pi i(j+1)k/N}) &= \mathrm{Re}\left[Ne^{4\pi ik/N}\left(\frac{1-e^{-2\pi ik/N}}{(1-e^{2\pi ik/N})^2}\right)\right] \\ &= \frac{N}{2}\end{aligned}$$

Hence

$$\begin{aligned} v_{N,k}(t) &= v^0_{N,k}(t) + \frac{\sqrt{2}c\tau}{\sqrt{N}}\binom{N}{2} \\ &= v^0_{N,k}(t) + \frac{\sqrt{N}c\tau}{\sqrt{2}} \end{aligned}$$

Similarly, for the w attractor coordinate

$$\begin{aligned} w_{N,k}(t) &= \frac{\sqrt{2}}{\sqrt{N}}\sum_{j=0}^{N-1}\sin(2\pi(j+1)k/N)x_{N,j}(t) \\ &= w^0_{N,k}(t) + \frac{\sqrt{2}c}{\sqrt{N}}\sum_{j=0}^{N-1}\sin(2\pi(j+1)k/N)(t-j\tau) \\ &= w^0_{N,k}(t) + \frac{\sqrt{2}ct}{\sqrt{N}}\sum_{j=0}^{N-1}\sin(2\pi(j+1)k/N) - \frac{\sqrt{2}c\tau}{\sqrt{N}}\sum_{j=0}^{N-1}j\sin(2\pi(j+1)k/N) \end{aligned}$$

where $w^0_{N,k}(t)$ is the w coordinate generated from the signal $x_0(t)$. We can express $\sin(2\pi(j+1)k/N) = -\frac{i}{2}\left(e^{2\pi i(j+1)k/N} - e^{-2\pi i(j+1)k/N}\right)$. As before, the t coefficient sums to zero and for the constant term, we have

$$\begin{aligned} \sum_{j=0}^{N-1}j\sin(2\pi(j+1)k/N) &= -\frac{i}{2}\sum_{j=0}^{N-1}j(e^{2\pi i(j+1)k/N} - e^{-2\pi i(j+1)k/N}) \\ &= \mathrm{Im}\left[Ne^{4\pi ik/N}\left(\frac{1-e^{-2\pi ik/N}}{(1-e^{2\pi ik/N})^2}\right)\right] \\ &= -\frac{N\sin(2\pi k/N)}{2(1-\cos(2\pi k/N))} \end{aligned}$$

Therefore

$$w_{N,k}(t) = w^0_{N,k}(t) + \frac{\sqrt{N}c\tau\sin(2\pi k/N)}{\sqrt{2}(1-\cos(2\pi k/N))}$$

We note that these results are consistent with the results derived in the paper for $(N,k) = (3,1)$.

Thus, the baseline drift results in a constant translation in v and w with the direction of movement $\theta_{N,k}$ defined by

$$\begin{aligned}
\tan\theta_{N,k} &= \frac{\sqrt{N}c\tau\sin(2\pi k/N)}{\sqrt{2}(1-\cos(2\pi k/N))}\Bigg/\frac{\sqrt{N}c\tau}{\sqrt{2}} \\
&= \frac{\sin(2\pi k/N)}{1-\cos(2\pi k/N)} \\
&= \frac{2\sin(\pi k/N)\cos(\pi k/N)}{2\sin^2(\pi k/N)} \\
&= \cot(\pi k/N) \\
&= \tan(\pi/2-\pi k/N)
\end{aligned}$$

Hence

$$\theta_{N,k} = \frac{\pi}{2}-\frac{\pi k}{N},\quad -\frac{\pi}{2}-\frac{\pi k}{N}$$

The shift in $v_{N,k}(t)$ is positive when $c>0$ which corresponds to the first of these. The magnitude of the shift from $(v^0_{N,k}, w^0_{N,k})$ is proportional to the slope c of the drift.

REFERENCES

Lyle J, Aston P. Symmetric Projection Attractor Reconstruction: Embedding in higher dimensions. *Chaos* **31** (2021) 113135.
